# Presence and distribution of dental enamel defects, recurrent aphthous lesions and dental caries in children with celiac disease

**DOI:** 10.12669/pjms.313.6960

**Published:** 2015

**Authors:** Kenan Cantekin, Duran Arslan, Ebru Delikan

**Affiliations:** 1 Kenan Cantekin, DDS, PhD, Assistant Professor, Department of Pediatric Dentistry, Faculty of Dentistry, Erciyes University, Kayseri, Turkey; 2 Duran Arslan, MD, Professor, Department of Pediatric Disease and Health, Subdivision of Pediatric Gastroenterology, Faculty of Medicine, Erciyes University, Kayseri, Turkey; 3 Ebru Delikan, DDS, Research Assistant, Department of Pediatric Dentistry, Faculty of Dentistry, Erciyes University, Kayseri, Turkey

**Keywords:** Celiac Disease, Dental caries, Enamel defects, Recurrent aphthous stomatitis

## Abstract

**Objective:**

To determine presence and distribution of enamel defects, recurrent oral aphthous lesions (RAS) and dental caries in children with Celiac Disease (CD) and compare the results with a healthy control group.

**Methods:**

Twenty- five CD patients age between 4- 16 years with no other systemic disease, were examined in Pediatric Gastroenterology Clinic of Erciyes University, Faculty of Medicine (Kayseri, Turkey) and then referred to Department of Pediatric Dentistry, Faculty of Dentistry for dental examination and treatment. The control group (25 patients) consisted healthy patients referred to the Department of Pediatric Dentistry, Faculty of Dentistry, Erciyes University for restorative treatment. Both the CD group and control group was examined by the same investigator for the following; (1) enamel defects, (2) recurrent aphthous stomatitis, (3) dental caries.

**Results:**

The mean dmft values for the CD group and control group were 3.25±3.25 and 4.56±2.87 respectively. The difference was not statistically significant.(*P*>0.05). The mean DMFT values for the CD and the control group were 3.75±2.62 and 1.83±1.7, respectively. There was a significant difference between the two groups (*P*<0.01). The prevalence of enamel defects and recurrent apthous stomatitis (RAS) was greater in celiac patients than in the control group. Enamel defects (in at least one permanent tooth) were observed in 12 out of 25 (48%) children in the CD group and four out of 25 children (16%).(*P* =0.01). Recurrent apthous stomatitis was found in 11/25 (44%) CD group, while no RAS was detected in the control group.

**Conclusion:**

Celiac Disease (CD) has adverse effects on oral health in term of enamel defect, recurrent aphthous stomatitis and caries score. Pediatricians and dentists especially pediatric dentists should be knowledgeable about oral symptoms of CD. Increased awareness can provide an early diagnosis and prevent long- term complications of this disease. On the other hand, further comprehensive investigations of CD patients can add to our understanding of the efficacy of CD on oral health in children.

## INTRODUCTION

Celiac disease (CD) is a common autoimmune chronic disorder related to permanent intolerance to the polypeptide fragments of gluten, a protein contained in some cereals, such as wheat, rye, and barley and can also be defined as gluten- sensitive entheropathy.^1^ Most common symptoms of the CD are; pain and discomfort in the digestive tract, chronic constipation and diarrhea, failure to thrive (in children), anemia and fatigue.^1^ On the other hand, malabsorption from the small intestine because of atrophy in mucosa of intestine, results in vitamin deficiencies in patients with celiac disease.^2^

The prevalence of CD is approximately 1% in the general population for American and European communities.^3^ Similarly, the ratio of CD was reported as 1:115 in Turkey.^4^ Patients with CD can also show some symptoms in oral cavity as it is a part of gastrointestinal system.^5^ The most common oral symptoms are recurrent aphthous stomatitis (RAS), dental enamel defects, delayed eruption, atrophic glossitis and angular chelitis.^5^

The aim of this study was to investigate the presence and distribution of enamel defects, oral aphthous lesions and decay- missing- filled teeth scores for primary teeth (dmft) and permanent teeth (DMFT) in children with CD and to compare the results with a healthy control group.

## METHODS

Twenty five CD patients between 4 - 16 years of age, and without any other systemic disease were selected for the study. The children were first examined in Pediatric Gastroenterology Clinic of Erciyes University, Faculty of Medicine (Kayseri, Turkey) and then referred to Department of Pediatric Dentistry, Faculty of Dentistry, Erciyes University for dental/oral management. The control group (25 patients) consisted of healthy patients referred to the Department of Pediatric Dentistry for restorative treatments.

This study was approved by the Ethical Committee of Erciyes University. An informed consent was obtained from parents of all the selected children. Both celiac patient group and the control group were examined by the same investigator for enamel defects, RAS and dmft/DMFT scores.

### Statistical analyses

Kolmogorov–Smirnov test was used to study the distribution of the parameters. The t-test for independent measurement was used for comparison of the dmft/DMFT scores between the control group and CD group. The χ^2^ test was used for comparison of the ratio of dental enamel defects between the control groups and the CD group. All statistical analyses were performed using SPSS Version 17 (SPSS Inc., Chicago, Illinois, United States of America). Statistical significance was set at 5%.

## RESULTS

Sixty one children were examined. Because of having another systemic disease (diabetes mellitus, thyroid disease, and dermatitis herpetiformis), eleven children were excluded from the study. Consequently 25 children with CD and 25 healthy children were included in the study.

The ratio of male- female in CD group was 10:15 and in control group 11:14. The age range of children in this study was between 4-16 years. The mean age was 8.94±2.08 years and 9.66±4.26 years in CD group and control group, respectively. There was no significant difference in terms of age and gender between CD group and control group (*P*>0.05) (Table-I).

**Table-I:** Demographics of Celiac Disease (CD) group and Control group.

			Gender	
	N	Mean age (years)	Girls	Boys
CD Group	25	8.94±2.08	15	10
Control Group	25	9.66±4.26	14	11
Total	50	9.30±3.23	29	21

The mean dmft values for the CD group and control group were 3.25±3.25 and 4.56±2.87, respectively. The difference was not statistically significant (*P*>0.05). The mean DMFT values for the CD and the control group were 3.75±2.62 and 1.83±1.7, respectively. There was a significant difference between the two groups (*P*<0.01) (Table-II).

**Table-II:** The mean dmft/DMFT score in CD children and healthy control group.

	CD group (n: 25)	Control group (n: 25)	P value
Mean dmf score	3.25±3.25	4.56±2.87	0.19
Mean DMF score	3.75±2.62	1.83±1.78	0.001

In the present study, the prevalence of enamel defects and recurrent aphthous stomatitis (RAS) was greater in celiac patients than in the control group. Enamel defects (in at least one permanent tooth) were observed in 12 out of 25 (48%) children in the CD group and four out of 25 healthy children (16%) (Table-III). The difference between the two groups was statistically significant (*P*=0.01). The enamel defects were generally present in anterior teeth.

**Table-III:** Oral manifestations in CD group and Control group.

	CD group (n: 25)	Control group (n: 25)	
RAS	11(44%)	0 (0%)	
Enamel Defects	12 (48%)	4(16%)	P=0.01

Recurrent apthous stomatitis (RAS) was found in 11/25 (44%) CD group, while no RAS was detected in the control group (Table-III).

## DISCUSSION

Several studies have been carried out comparing DMFT/dmft scores and dental enamel defects between the CD children and healthy control groups.^1^,^2^,^9^ Avşar and Kalaycı^2^ found that the number of caries-free subjects in the control group was higher (38%) than in the CD group (17%). In accordance with this study, we found higher mean DMFT score in CD group than the control group. On the other hand Acar et al.^10^ found that mean DMFT/dmft scores were not significantly different between CD and healthy control group.

The cause of dental enamel defects in Celiac patients is not exactly clear.^3^,^11^,^12^ Malabsorption of various macro- micro nutrients (iron, calcium folate and fat soluable vitamins) may be responsible for dental enamel defects.^13^,^14^ Dental enamel defects reflect the period of interruption of amelogenesis.^15^ Aine et al. and Maki et al. defined dental enamel defects as an autoimmune response of the enamel organ.^15^,^16^ In a study carried out by Ertekin et al.^17^ celiac-type dental enamel defects defined as ’systematic’ because they were symmetrical and could be seen in all sections of dentition. In clinical identification of CD, the presence of higher incidence of enamel defects in permanent teeth could be a fundamental clue for clinicians. The overall prevalence of dental enamel defects in celiac patients ranges from 9.52% to 95.94% (mean value of 51.12%). In a study, Avşar and Kalayci^2^ mentioned that enamel defects were significantly higher in CD patients (42.2%) than healthy control group (9.4%). In the study of Wierink et al. enamel defects were found in 55% of CD patients and 18% of the control group.^12^ In agreement with these studies, the present study also showed higher prevalence of enamel defects (48%) than the control group(16%) (Table-III). The enamel defects in the present study were generally symmetrical and mostly seen in anterior teeth.

Recurrent apthous stomatitis (RAS) is a common oral mucosal disease. It is characterized by one or several discrete, shallow, painful ulcers on the unattached mucous membranes. The reason for RAS is idiopathic but it is generally related with iron, folate and vitamin B12 deficiencies, stress, allergies, trauma, hormonal imbalance and infections.^18^,^19^ When there is a recurrent RAS in a child, it should be evaluated as a high probability CD patient and should be examined accordingly. In CD patients the prevalence of RAS ranges from 9.66% to 40.98% (mean value 20%).^3^,^19^ The present study confirmed that that CD patients especially before starting gluten- free diet have higher RAS prevalence (44%) than healthy control group (0%) (Table-III). Statistically significant difference was found between CD and control groups. Similarly, Procaccini et al.^1^ also showed that RAS prevalence was higher in CD patients in comparison with the controls.

Pediatricians and dentists especially pediatric dentists should be knowledgeable about oral symptoms of Celiac Disease. Increased awareness can provide an early diagnosis and prevent long- term complications of this disease. The dentist could be the first specialist identifying Celiac Disease, especially “atypical” CD.

## CONCLUSIONS

1. The prevalence of dental enamel defects was higher in CD children than in healthy children.
2. The DMFT scores were higher in CD children as compared to control group.
3. RAS was frequently observed in CD children especially before starting gluten-free diet.
4. Further comprehensive investigations of CD patients can add to our understanding of the efficacy of CD on oral health in children.
